# Cognitive Memory Comparison Between Tinnitus and Normal Cases Using Event-Related Potentials

**DOI:** 10.3389/fnint.2018.00048

**Published:** 2018-10-12

**Authors:** Abdoreza Asadpour, Ali Alavi, Mehran Jahed, Saeid Mahmoudian

**Affiliations:** ^1^Department of Electrical Engineering, Sharif University of Technology, Tehran, Iran; ^2^ENT and Head & Neck Research Center, Iran University of Medical Sciences (IUMS), Tehran, Iran

**Keywords:** tinnitus, electroencephalogram, cognitive memory, event-related potential, p300

## Abstract

About 20 percent of people above 60 years old suffer from tinnitus though no definitive treatment has been found for it. Evaluation of electrical brain activity using Event-Related Potentials (ERPs) is one of the methods to investigate the underlying reasons of tinnitus perception. Previous studies using ERPs suggest that the precognitive memory in tinnitus groups is negatively affected in comparison to the normal hearing groups. In this study, cognitive memory has been assessed using visual and auditory P300 response with oddball paradigm. Fifteen chronic tinnitus subjects and six normal hearing subjects participated in the experiment. *T*-test with significance level of 0.05 was applied on amplitude and latency of auditory and visual P300 for all electroencephalography (EEG) channels separately to compare tinnitus and normal hearing groups where the tinnitus group showed meaningful lower amplitude of auditory P300 peak in three EEG channels.

## Introduction

Tinnitus is an underlying condition that includes ringing, buzzing, whistling, or hissing in the ears in the absence of an external stimulus such as sound (Rewar, 2015). In the US, it is estimated that about 25.3% of adults have some form of tinnitus (Shargorodsky et al., 2010), and about 5–10 percent of adults have chronic tinnitus (Henry et al., 2015). The impact of tinnitus can be extremely disturbing and debilitating, markedly impairing mood, concentration, functioning and overall quality of life in approximately 1–3 percent of the adult population (Adrian and El Refaie, 2000). The relatively high prevalence of tinnitus in the population makes it a common problem that can be classified broadly into two main types: objective and subjective. Subjective tinnitus is defined as the false perception of sound only experienced by the individual (Snow, 2004). Subjective tinnitus is the most frequently occurring type of tinnitus and results from exposure to loud noise, anticholinergic effects of some medications, increased age, or, more commonly, it has no identifiable cause aside from hearing loss, which is often comorbid (Lockwood et al., 2002).

Biological markers of brain functioning related to symptom pathology are often studied to inform more specific treatment plans for patients. Electroencephalography (EEG) was developed in the late 1800s as a device capable of measuring electrical activity produced by the brain from electrodes placed on the scalp. The electrical activity, commonly referred to as brain waves, is produced from many neurons firing within the brain (Niedermeyer and da Silva, 1999). In addition to assess brain wave production, EEG also measures a phenomenon known as *evoked potentials*, including the auditory P300 waveform, which is the main focus of the current study. There are two main classifications of evoked potentials: *exogenous* and *endogenous potentials*. Exogenous potentials are dependent on features of the stimuli, such as the brightness of a light, or loudness of a tone. Essentially, exogenous potentials are the brain’s acknowledgment of a stimuli’s existence. Endogenous potentials, also known as event-related potentials (ERPs), are evoked when the tested individual is required to make meaning of, or a decision about, an incoming stimulus. ERPs are electrical impulses that are generated by the production of thoughts and perceptions occurring in response to an internal or external stimulus (Coles and Rugg, 1996).

The P300 ERP is a positive occurring potential in frontal, central, temporal and parietal region of scalp based on the tip of nose as reference, and the latency of the waveform occurs at approximately 300 ms after the presentation of a stimulus presentation. Previous studies have suggested that P300 amplitude is a measure of attentional allocation and focus and it is thought to be effected by various clinical presentations and diagnoses, such as dementia, depression, schizophrenia, bipolar disorder and ADHD, among others. P300 latency is believed to measure how quickly one can process attentional information. P300 latencies longer than 300 ms could suggest slower attentional processing speed (Polich, 1991; Polich and Kok, 1995).

There are various studies on attentional dysfunctions in different diseases using P300. A research on 16 persons who presumed to be in early stages of Alzheimer’s disease and 16 normal persons as control group was conducted using oddball paradigm. P300 latency in the first group was higher in comparison to control group (Polich et al., 1990). Another study investigated the effects of detoxification and buprenorphine treatment on opiate and cocaine users. P300 amplitude was higher in patients with buprenorphine treatment in comparison to control group (Kouri et al., 1996). Assessment of P300 in schizophrenia patients was the research which conducted on 24 patients and 24 normal control group. Auditory and visual P300 were presented to subjects. Results showed that P300 latency is a better feature to separate patients and control group (Neuhaus et al., 2013). In 2013, a study implemented on 28 chronic tinnitus subjects and 33 normal hearing control group using mismatch negativity (MMN) as a type of ERPs to assess pre-attentive processes between two groups and found lower amplitude of MMN peak in tinnitus subjects. This study suggested that precognitive memory in tinnitus group has dysfunction in comparison to the normal hearing groups (Mahmoudian et al., 2013). Precognitive memory dysfunction may suggest that the cognitive memory in tinnitus group may indicate deficiencies compared to normal hearing group. In another study, a top-down analyses using oddball paradigm and bottom-up analyses using passive listening paradigm were conducted on 15 tinnitus patients without hearing loss and 15 healthy volunteers. They selected six EEG electrodes (Cz, CP1, CP2, Pz, P3 and P4) for evaluating P300 and found significant decrease of P300 peak amplitude in tinnitus group especially in Cz channel. Also they found no significant differences in latency of P300 between two groups and suggested a top-down impairment in tinnitus patients without hearing loss (Hong et al., 2016). Cognitive impairment in chronic tinnitus subjects was evaluated using oddball paradigm to evoke P300 between 95 patients having mild tinnitus and 112 patients with severe tinnitus. ERPs were recorded in three channels (Fz, Cz and Pz) and a positive correlation was found between P300 latencies and tinnitus severity (Wang et al., 2018).

Previous studies have not found a common and conclusive result regarding the impairment of cognitive memory in tinnitus subjects. In this study, auditory and visual cognitive memory have been evaluated to compare tinnitus and normal hearing subjects using visual and auditory P300 responses. The following sections contain a brief theoretical background of the methods which were used in the study, materials and methods of the study, results of the proposed methods and discussion.

## Theoretical Background

### Problem Specification and Assumptions

As explained in “Introduction” section, pre-attentive malfunction has been reported in tinnitus subjects. This study focuses on assessment of attentional allocation in tinnitus subjects. Finding and investigating a parameter which attentional dysfunction can be extracted from will be of importance. Previous studies suggest that P300 is an index of cognitive and attentional processing (Polich, 1991) and studying P300 changes on tinnitus patients can provide essential information about underlying mechanisms of tinnitus.

Many types of stimulus changes can elicit P300, including visual and auditory stimulations. The measure used in this study is the oddball paradigm. This task involves presenting the patient with standard and non-standard, or odd tones (Polich, 2007). Typically, the paradigm consists of 140–180 total trials. Most of the presentations or 80% of all trials are of a standard tone with few presentations of the odd tone. The odd tone is presented in a higher frequency than standard tone presentations. P300 is considered an ERP and its analytical methods are therefore similar.

### Signal Averaging

A major limitation of the use of ERPs is that their amplitude is very small relative to the ongoing amplitude of the EEG. ERPs are thus not generally visible within a segment of EEG corresponding to the presentation of a single stimulus or the occurrence of a single event (Picton et al., 1995). The ongoing background EEG (activity not related to the eliciting event) is essentially random. On the other hand, it is assumed that the response to the stimulus is constant. Over an infinite number of trials, the average of a constant is the constant itself. Experimental paradigms employing ERPs are thus often designed to elicit the response of interest many times so that individual responses to stimuli can be summated (Luck, 2005).

### Baseline Correction

Though the averaging process reduces the amount of background noise, it is impossible to remove this noise entirely. The period prior to the onset of the stimulus can be used as an estimate of noise remaining in the averaged ERP. This pre-stimulus interval should appear relatively flat, if most of the background noise has been reduced sufficiently through the averaging process. For this reason, the pre-stimulus interval is also used to provide a baseline, approximating zero voltage from which the ensuing responses are measured (Woodman, 2010).

## Materials and Methods

### Experimental Design

#### Auditory Stimuli

Three-hundred auditory stimuli were presented to the participants pseudo-randomly in three blocks and there was a several seconds silent gap between each two blocks. Each block contained 80 standard stimuli which were 4 kHz single-tone sounds with a duration of 150 ms, 10 rare stimuli as targets that were narrow-band sounds around 6 kHz frequency (nearly similar to the frequency of tinnitus in most tinnitus subjects) with the same duration as standard stimuli and 10 novel sounds which were environmental sounds like the sound of water or animals with a duration of 200–300 ms with the intensity of 70 dB SPL to force the participants to concentrate more. A silent gap of 1,400–1,800 ms was placed between each of the two stimuli and each stimulus along with its immediate silent gap was considered as an epoch.

#### Visual Stimuli

Two-hundred visual stimuli containing 160 blue triangles as standard and 40 yellow circles as target stimuli were shown to participants in two 100 stimuli blocks pseudo-randomly with a duration of 300 ms followed by a 1,200–1,600 ms of black display (as an epoch) for each stimulus after the auditory stimulation phase.

#### Presenting the Stimuli

**Figure 1:**
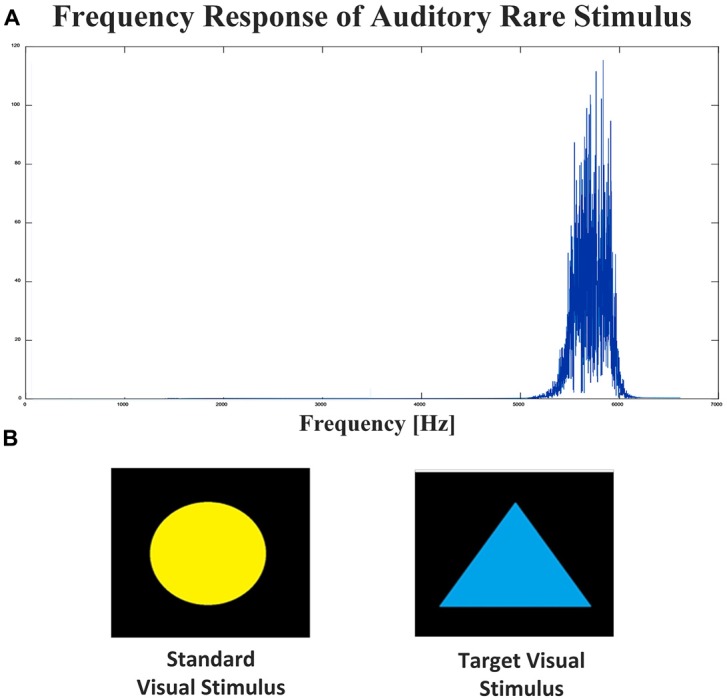
Visual and auditory stimuli. **(A)** Frequency response of auditory rare stimulus. **(B)** Standard and target (Rare) visual stimulus.

Figure 1 shows the frequency response of auditory rare stimulus and standard and Target Visual stimuli. Targets were not presented consecutively and at least one standard stimulus was inserted between each two targets. During the oddball paradigm, the patient is instructed to click a computer mouse during rare stimulus presentations only. Participants are asked to do nothing in response to the standard tone. Attending to the rare stimulus serves to elicit the P300 response, as the oddball paradigm serves as a cognitive decision making task.

### Subjects

Twenty-one individuals consisting of 15 persons (10 male, five female and 39 ± 11 years old) in tinnitus group and six persons (three male, three female and 27 ± 5 years old) in normal hearing group participated in this study. All participants were right-handed and had normal hearing.

### Acquisition Protocol

All participants sat in a comfortable chair in an electrically attenuated booth watching a video about nature without audio and with Persian subtitles. Two speakers situated at about 45-degree angles were used to present the stimuli to the subject. Participants were instructed to minimize eye movements and blinking. In addition, subjects were required to maintain complete muscle relaxation of the hands during all data acquiring periods.

Subjects wore a 32 Ag/AgCl EEG electrode cap with tip of nose as reference electrode. Standard 10-20 EEG system and EOG recording procedures were employed. Horizontal and vertical EOG channels affixed to the left canthi and the lower orbital ridge documented eye movements. Impedances of all electrode channels were kept below 10 kΩ with reference to Tip of Nose. Sampling frequency was 1 kHz and the signal and band-pass filtered at 0.4–200 Hz where a notch filter of 50 Hz was applied to remove the power supply noise. The auditory and visual stimuli were applied to subjects using Presentation software (Neurobehavioral Systems) and stimulus onset was automatically documented with inputted markers within Presentation file and as a pulse trigger in one of the channels.

### Pre and Post Processing and Analysis

The recorded data was imported to MATLAB 2013a and filtered using a bandpass finite impulse response (FIR) filter with the frequency of 0.5–10 Hz and artifacts, including EOG, ECG and high frequency components, were removed by ICA. ICA algorithm used in this study was Runica and removed components were selected manually by the user. The FIR filter and ICA were created by EEGLAB toolbox for MATLAB (Delorme and Makeig, 2004).

Target signals were calculated by averaging target epochs and P300 peak and its latency from the onset of target stimulus were extracted for each channel using local peak detection algorithm. To avoid serious errors caused by unexpected noise and artifacts, epochs for which the maximum level or the mean energy of the signal was higher than a threshold or the subject did not respond by clicking computer mouse were removed from the analysis. This threshold varied among subjects and about 25 percent of target epochs were removed per subject.

## Results

**Figure 2:**
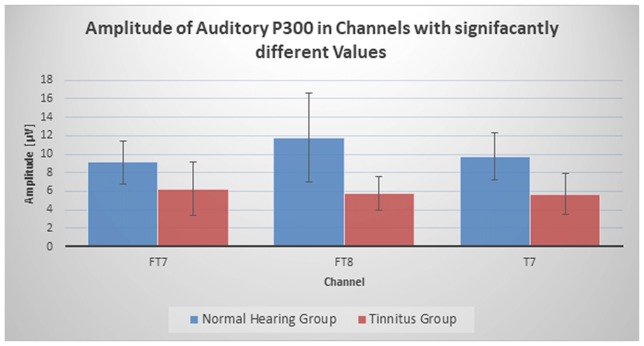
Amplitude of auditory P300 in three channels with significantly different values.

**Figure 3:**
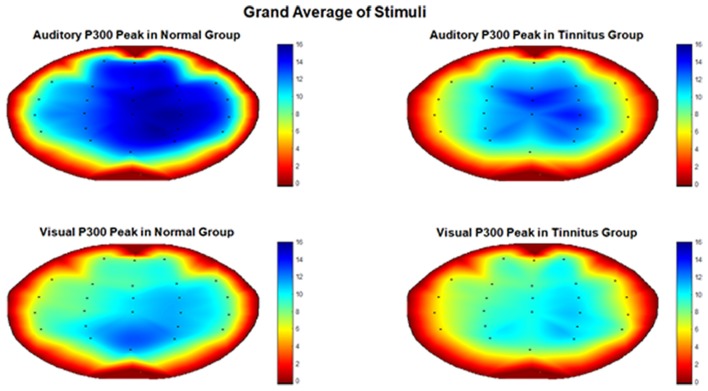
Topographic map of the grand average of P300 peak amplitude in Normal Hearing and Tinnitus group.

Visual and Auditory P300 peak amplitude and latency were calculated for all channels and each subject under the supervision of a neuroscientist. To investigate if tinnitus group and normal hearing group have meaningful difference, each channel was compared between groups separately using a Two-Sample Assuming Unequal Variances *T*-test with significance level of 0.05. Results showed that no channel had significant difference in P300 latency and three channels (FT7 (*p* < 0.045), FT8 (*p* < 0.025) and T7 (*p* < 0.01)) in auditory P300 peak had notable difference between tinnitus and normal hearing group. Figure 2 shows the difference between two groups in these channels. The P300 peak in these channels were lower in tinnitus group in comparison to normal hearing group. To check the null hypothesis of P300 peak amplitude of auditory stimulus in all channels between Normal Hearing group and Tinnitus group, a Randomization (Permutation) test of two independent samples on sample means with 2,000 iterations was implemented on all channels with significance level of 0.05 and the result rejected the null hypothesis (*p* < 0.0001). Also, Randomization of two independent samples on sample means with 2,000 iterations was implemented on the three channels, which had meaningful differences in *T*-test, with significance level of 0.05 and results showed significant differences between two groups (FT7 (*p* < 0.047), FT8 (*p* < 0.0005) and T7 (*p* < 0.004)). The randomization analysis was generated using the Real Statistics Resource Pack software (Release 5.4, Copyright (2013–2018) Charles Zaiontz^1^). Table 1 represents the grand average of all channels in Normal Hearing and Tinnitus group. Also, Figure 3 shows the topographic map of the grand average of P300 peak amplitude in two groups for auditory and visual stimulus.

**Table 1:** Grand average of all channels in normal hearing and tinnitus group.

Stimulus		Normal Hearing group	Tinnitus group	*P*-value
Auditory P300	Peak latency (ms)	380 ± 12	376 ± 24	0.66
	Peak amplitude (μV)	13 ± 5	10 ± 3	0.25
Visual P300	Peak latency (ms)	394 ± 38	390 ± 31	0.82
	Peak amplitude (μV)	9 ± 4	8 ± 2	0.55

## Discussion

This study evaluated the effect of tinnitus on cognitive memory in 15 subjects suffering from chronic subjective tinnitus comparing to six normal hearing subjects by recording peak and latency of their Auditory and Visual P300. *T*-test with significance level of 0.05 was used on peak and latency of Visual and Auditory P300 to compare tinnitus and normal group in all channels and the tinnitus group showed meaningful lower amplitude in three channels for auditory P300 peak. The decrease in amplitude of auditory peak is an indicator of lower focus and attention especially for sounds near the frequency of tinnitus (Polich, 1991; Polich and Kok, 1995). This result is in accordance with a previous study that used oddball paradigm to elicit auditory P300 (Hong et al., 2016) and may suggest an impairment of auditory cognitive memory in tinnitus subjects. We found no significant difference in visual P300 time characteristics between two groups which may indicate that visual cognitive memory is not affected in tinnitus subjects. These findings may improve the understandings of cognitive memory impairment in tinnitus patients. However definitive conclusion about the negative effect of tinnitus on cognitive memory was not observed and may require further investigation.

### Changing Visual Stimulus

**Figure 4:**
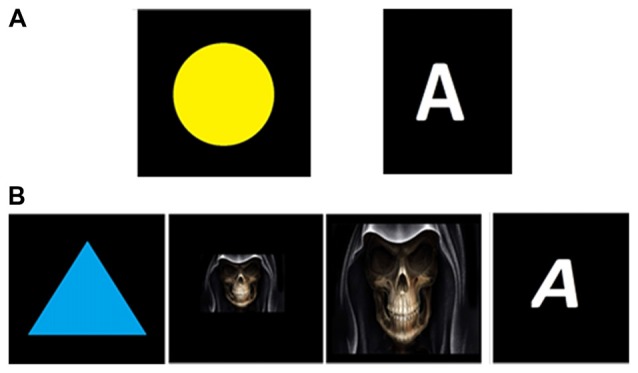
Visual standard and rare (Deviant) stimuli of all four pairs in changing visual stimulus session. **(A)** Standard stimuli, **(B)** Rare (Deviant) stimuli.

**Figure 5:**
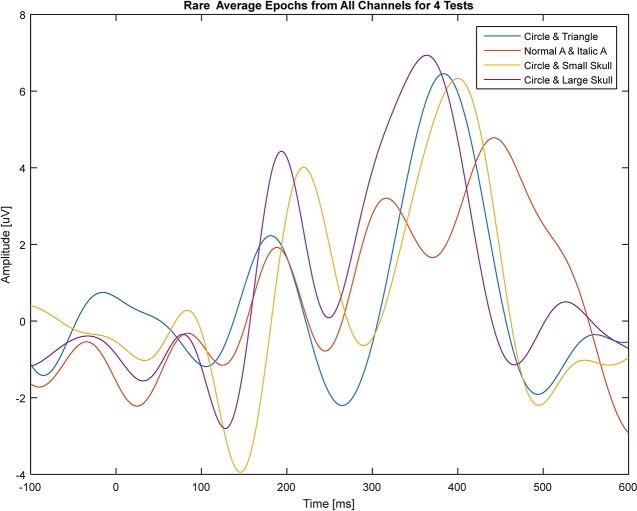
Averaged epoch of all four pairs in all channels (Blue: Circle and Triangle, Red: Normal A and Italic A, Yellow: Circle and Small Skull and Violet: Circle and Large Skull).

To investigate the effect of changing the visual stimulus on peak and latency of Visual P300, four different pair of frequent and rare stimuli were presented to a normal hearing 30 years old male subject. A yellow circle as standard and a blue triangle, a big and small skull as rare stimulus with Normal A as standard and Italic *A* as rare stimulus were applied to the subject. Figure 4 shows these pair of stimuli. Figure 5 shows the averaged epoch of all four pairs in all channels. The latency of P300 differs for each pair of stimulus and the large skull caused the earliest P300.

This shows that the latency and peak of P300 depend on several parameters and may suggest that each subject could respond to same pair of stimulus differently and cause insignificant difference in visual P300 time characteristics between tinnitus and normal hearing group. However, this aspect of our results may require further investigation in order to be confirmed.

## Ethics Statement

This study was approved by the Ethics Committee of Iran University of Medical Sciences (IUMS; ENT and Head & Neck Research Center), through Ethics Code of IR.IUMS.REC1396.33174. Furthermore, participants provided written informed consent. All tinnitus and normal subjects completed consent form that allowed us to conduct an EEG data acquisition.

## Author Contributions

ABA contributed to design of the work, data acquisition, analysis of data and draft of the manuscript. ALA contributed to design of the work, data acquisition, analysis of data and draft of preliminary results. MJ contributed to the concept and design of the work, supervising the analysis and revising and finalizing the manuscript. SM contributed to the concept and design of the work, supervision of experiments and revising the manuscript.

## Conflict of Interest Statement

The authors declare that the research was conducted in the absence of any commercial or financial relationships that could be construed as a potential conflict of interest.

## Footnotes

^1^www.real-statistics.com
